# Antioxidant Activity of *Gracilaria lemaneiformis* Polysaccharide Degradation Based on Nrf-2/Keap-1 Signaling Pathway in HepG2 Cells with Oxidative Stress Induced by H_2_O_2_

**DOI:** 10.3390/md20090545

**Published:** 2022-08-24

**Authors:** Xiaoshan Long, Xiao Hu, Chuang Pan, Huan Xiang, Shengjun Chen, Bo Qi, Shucheng Liu, Xianqing Yang

**Affiliations:** 1 College of Food Science and Technology, Guangdong Ocean University, Guangdong Provincial Key Laboratory of Aquatic Product Processing and Safety, Guangdong Province Engineering Laboratory for Marine Biological Products, Guangdong Provincial Engineering Technology Research Center of Seafood, Key Laboratory of Advanced Processing of Aquatic Product of Guangdong Higher Education Institution, Zhanjiang 524088, China; 2 Key Laboratory of Aquatic Product Processing, Ministry of Agriculture and Rural, South China Sea Fisheries Research Institute, Chinese Academy of Fishery Sciences, Guangzhou 510300, China; 3 Co-Innovation Center of Jiangsu Marine Bio-Industry Technology, Jiangsu Ocean University, Lianyungang 222005, China; 4 Collaborative Innovation Center of Seafood Deep Processing, Dalian Polytechnic University, Dalian 116034, China

**Keywords:** *G.* *lemaneiformis*, polysaccharide, degradation, free radicals, antioxidant, signaling pathway

## Abstract

The objective of this research was to investigate the antioxidant activity of *Gracilaria* *lemaneiformis* polysaccharide degradation and its underlying mechanism involved in the Nrf-2/Keap-1 signaling pathway in HepG2 cells with oxidative stress induced by H_2_O_2_. The result of the scavenging ability of free radicals showed that GLP-HV (polysaccharide degraded by H_2_O_2_–vitamin C (Vc)) performed a better scavenging ability than GLP (*G.* *lemaneiformis* polysaccharide). Moreover, the scavenging ability of polysaccharide to these free radicals from strong to weak was as follows: superoxide radical, ferric ion, ABTS^+^, and DPPH radical, and their IC_50_ values were 3.56 ± 0.0028, 4.97 ± 0.18, 9.62 ± 0.35, and 23.85 ± 1.78 mg/mL, respectively. Furthermore, GLP-HV obviously relieved oxidative stress in HepG2 cells, which strengthened the activity of T-AOC, CAT, GSH-PX, and SOD, and diminished the intensity of MDA, intracellular ROS, and calcium ion based on the Nrf-2/Keap-1 signaling pathway. The PCR result revealed that polysaccharide upregulated the expression of the genes Nrf-2, HO-1, NQO-1, and ZO-1 and downregulated Keap-1. The correlation between chemical properties and antioxidant mechanism of GLP-HV was evaluated via a heat map. The results illustrated that reducing sugar and active groups presented a positive correlation, and molecular weight and viscosity exhibited a negative relation with antioxidant activity.

## 1. Introduction

Oxidative stress refers to a state of imbalance between oxidation and antioxidant effects in the body, leading to neutrophil inflammatory infiltration, enhanced protease secretion, and the production of a large number of oxidative intermediates [1,2,3]. Oxidative stress is a negative effect caused by free radicals in the body and is considered to be an important factor in aging and chronic disease, including cancer, diabetes, and heart disease [4,5]. It is well known that antioxidants are commonly used in daily life to alleviate oxidative stress, including butyl-hydroxyanisole (BHA) and dibutyl-hydroxytoluene (BHT) and tert-butyl-hydroquinone (TBHQ), which have poor thermal stability and certain toxic and side effects on humans, which are not conducive to long-term use [6,7]. Thereupon, it is essential to analyze natural antioxidants to develop markets to mitigate oxidative stress.

A number of research studies have focused on plant and algal active substances with antioxidant activity attributed to their nontoxic side effects, including polysaccharide, polyphenols, tocopherol, and flavonoids [8,9,10]. *G. lemaneiformis* belongs to edible economic red algae, which is rich in polysaccharide with diverse physiological bioactivities [11]. Wen et al. [12] discovered that the polysaccharide of *G. lemaneiformis* enhances the expression of superoxide dismutase (SOD), glutathione peroxidase (GSH-PX), and catalase (CAT), reducing the level of malondialdehyde (MDA), which verified the antioxidant activity of GLP. Moreover, recent research studies have demonstrated that polysaccharide displays stronger physiological activity after degradation or modification [13,14]. Tang et al. [15] confirmed that *G. lemaneiformis* polysaccharide–selenium nanoparticles exhibit stronger antioxidant activity than *G. lemaneiformis* polysaccharide via scavenging free radicals. Polysaccharide treated with UV/H_2_O_2_ performed better biological activity in RAW 264.7 cells [16]. UV/H_2_O_2_ or other degradation methods destroy the glycosidic bonds of polysaccharide, reduce the molecular weight and viscosity of polysaccharide, and change the monosaccharide composition and active groups, thus enhancing the physiological activity of polysaccharide. Qiu et al. [17] verified that low-molecular-weight polysaccharide reinforced the CAT and GSH-PX activity and significantly enhanced the antioxidant activity in mice. In previous studies, GLP degraded by H_2_O_2_ and Vc elevated the better effects may be due to its lower molecular weight and monosaccharide compositions [18,19].

Increasing evidence has demonstrated that the antioxidant mechanisms are mainly mediated by nuclear factor erythroid 2-related factor 2 (Nrf-2) signaling pathways [20]. Nrf-2 is present in the cytoplasm associated with the negative regulatory protein Kelch-like ECH-associated protein 1 (Keap-1), which interacts with Nrf-2 and acts as an adaptor protein, maintaining Nrf-2 at a low level and allowing it to be continuously degraded by the proteasome in an ubiquitin-mediated process [21,22]. Nrf-2 has been acknowledged to play a pivotal role in oxidative stress response, which is involved in the regulation of multiple genes related to oxidative stress, and is related to numerous antioxidant mechanisms. The activation of Nrf-2 can restrain the expression of Keap-1 protein, an important regulator of cellular oxidative stress response, and stimulate the expression of downstream genes. Jiang et al. [23] investigated the antioxidant mechanism of Jiuzao tetrapeptide and discovered that inflammatory cytokines were suppressed through the upregulation of Nrf-2 and the downregulation of Keap-1.

To evaluate the changes of biological properties and antioxidant activity in vitro and in vivo from *G. lemaneiformis* polysaccharide before and after degradation, the content of 3,6-anhydrogalactose, active groups and viscosity, and free radical scavenging ability were investigated. Moreover, this study measured the antioxidant indexes (SOD, MDA, CAT, GSH-PX, ROS, calcium ion, and cell viability) and mRNA expression of related genes involved in the antioxidant signaling pathway in HepG2 cells. Furthermore, this research also aimed to explore the correlation between biological properties and antioxidant mechanism through a heat map.

## 2. Results

### 2.1. The Content of 3,6-Anhydrogalactose and Sulfate and Carbon Groups of GLP and GLP-HV

3,6-Anhydrogalactose belongs to one of the main components in *G. lemaneiformis* polysaccharide, which may be related to the biological activities [11]. Sulfate and carbon groups are the functional groups of polysaccharide that may improve its physiological activities to a certain extent. As shown in Table 1, the contents of 3,6-anhydrogalactose from GLP, GLP-HV, GLP-H, and GLP-V were 35.69 ± 0.34, 37.18 ± 0.48, 34.04 ± 0.77, and 43.98 ± 1.07%, respectively. The sulfate groups of GLP, GLP-HV, GLP-H, and GLP-V were 4.22 ± 0.08, 7.53 ± 0.0.05, 5.38 ± 0.25, and 5.48 ± 0.13%, respectively, which was consistent with the infrared results [18]. In addition, the contents of the carbon group determined in GLP, GLP-HV, GLP-H, and GLP-V were 3.31 ± 0.19, 4.22 ± 0.32, 4.32 ± 0.14, and 4.41 ± 0.02 μmol/g, respectively.

### 2.2. The Viscosity of Polysaccharides of G. lemaneiformis

**Figure 1:**
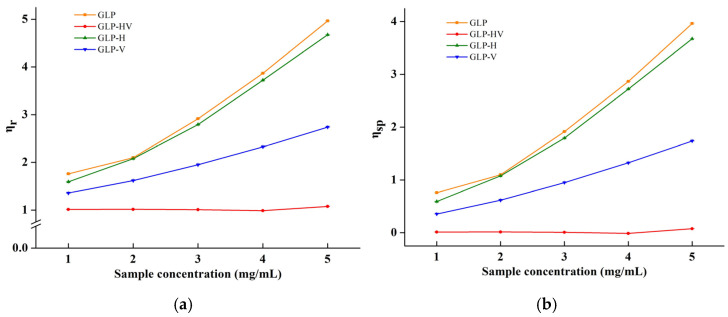

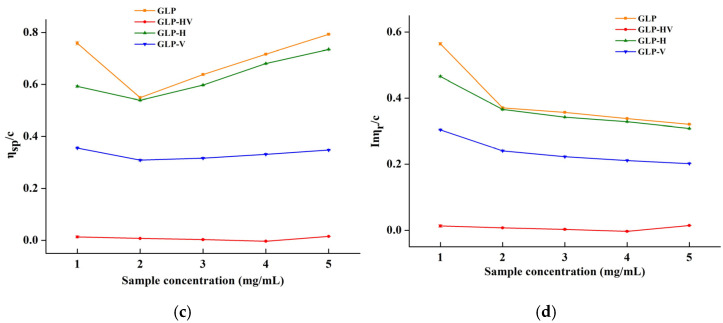
The viscosity analysis of *G. lemaneiformis* polysaccharide and its degradation products. Note: η0: viscosity of solvent; η: viscosity of solution; ηr: relative viscosity; ηsp: specific viscosity; ηsp/c: reduced viscosity; Inηr/c: inherent viscosity. (**a**) The relative viscosity of GLP, GLP-HV, GLP-H, and GLP-V; (**b**) the specific viscosity of GLP, GLP-HV, GLP-H, and GLP-V; (**c**) the reduced viscosity of GLP, GLP-HV, GLP-H, and GLP-V; (**d**) the inherent viscosity of GLP, GLP-HV, GLP-H and GLP-V.

Relative viscosity (ηr) is the viscosity of a solution relative to the concentration of the solvent, and its value is proportional to the solution viscosity. As shown in Figure 1a, the ηr value of GLP-HV was independent of the solution concentration, while the other three polysaccharides were dose dependent on concentration and viscosity. The ηr value of GLP-HV was close to “1” than its viscosity approach to the solvent. Specific viscosity (ηsp) reflects the internal friction effect between pure solvent and polymer, and its variation trend is the same as ηr, observed in Figure 1b. Reduced viscosity (ηsp/c) is present in Figure 1c; the variation trend of GLP, GLP-H, and GLP-V declined first and then increased, while GLP-HV almost remained stable (near to zero). The inherent viscosity (Inηr/c) of GLP-HV also remained unchanged with increasing concentration, whereas other three polysaccharides attenuated. All of these results illustrated that the viscosity of polysaccharide declined due to degradation, and Vc combined with H_2_O_2_ had the best effect.

### 2.3. Scavenging Ability of Polysaccharides of G. lemaneiformis on Free Radicals

**Figure 2:**
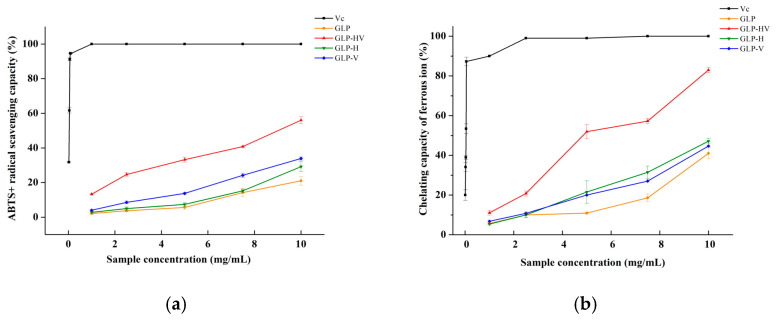

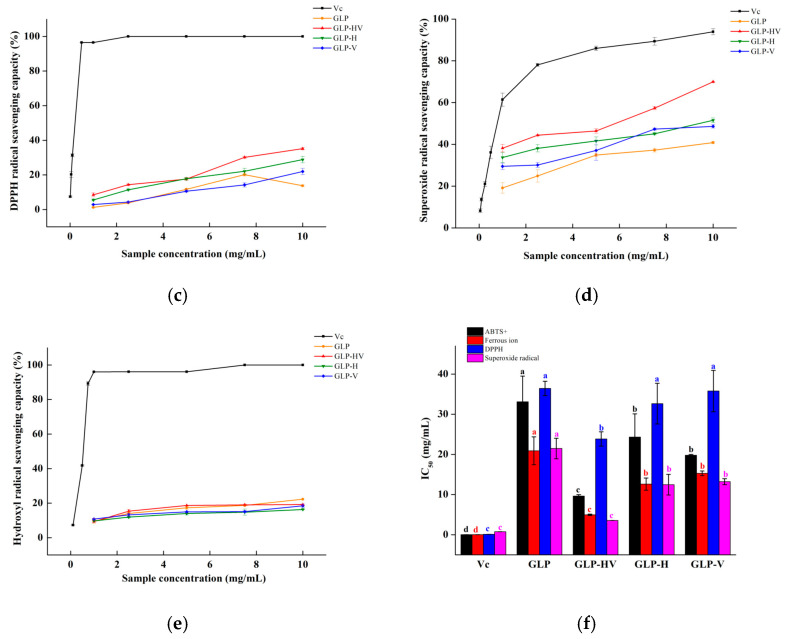
Scavenging ability of *G. lemaneiformis* polysaccharide and its degradation products. Note: (**a**) the ABTS^+^ scavenging activity of GLP, GLP-HV, GLP-H, and GLP-V; (**b**) the chelating capacity of ferrous ion of GLP, GLP-HV, GLP-H, and GLP-V; (**c**) the DPPH scavenging activity of GLP, GLP-HV, GLP-H, and GLP-V; (**d**) the superoxide radical scavenging activity of GLP, GLP-HV, GLP-H, and GLP-V; (**e**) the hydroxyl radical scavenging activity of GLP and GLP-HV. a, b, c and d in (**f**) represent a significant difference among groups on the same radical scavenging, and *p* < 0.05 indicates a significant difference.

Figure 2a–f displays the free radical scavenging capacity of polysaccharide before and after degradation. All of these four polysaccharides were dose dependent on the scavenging capacity of ABTS^+^, ferric ion, DPPH, and superoxide radical, except for the hydroxyl radical. As presented in Figure 2a, the ABTS^+^ scavenging capacity with concentration dependence was observed in all groups. At 10 mg/mL, the ABTS^+^ scavenging capacities of GLP, GLP-HV, GLP-H, and GLP-V were 21.01 ± 2.55, 56.01 ± 2.04, 29.19 ± 2.80, and 33.8 ± 0.88%, respectively. The IC_50_ values of GLP, GLP-HV, GLP-H, and GLP-V were calculated to be 33.09 ± 6.42, 9.62 ± 0.35, 24.31 ± 4.43, and 19.78 ± 0.21 mg/mL, respectively, which were significantly higher than that of Vc (0.029 ± 0.00058 mg/mL) (*p* < 0.05). Polysaccharide may provide electrons and hydrogen atoms to unstable ABTS^+^, thus achieving the goal of scavenging free radicals [24].

Ferrous ion chelating capacity refers to the reaction of ferric ion with other groups to generate chelates, converting ferric ion to ferrous ion. The scavenging ability of four kinds of polysaccharides on ferric ion was comparable to ABTS^+^. In Figure 2b, the chelating capacity of ferrous iron was observed in all groups with concentration dependence. The ferrous iron chelating abilities of GLP, GLP-HV, GLP-H, and GLP-V at 10 mg/mL were 41.02 ± 2.85, 82.93 ± 1.27, 47.12 ± 1.41, and 44.68 ± 2.43, respectively. The IC_50_ values of GLP, GLP-HV, GLP-H, GLP-V, and Vc were calculated by IBM SPSS software (version 22, Palo Alto, CA, USA) to be 20.89 ± 3.48, 4.96 ± 0.18, 12.60 ± 1.53, 15.28 ± 0.59, and 0.029 ± 0.001 mg/mL, respectively. GLP-HV expressed the best chelating ability of ferrous ion compared with the other polysaccharide (*p* < 0.05). It has been reported that the bridging structure formed between the carboxyl group and divalent ion in galacturonic acid may be the main reason for the metal ion chelating ability of polysaccharides and the sulfate group with high nucleophilic character as well as lower ferric ions in the complex by electron transfer reaction [25].

As presented in Figure 2c, the four polysaccharides have appreciable scavenging ability on DPPH radical that also emerged the phenomenon of concentration dependence. GLP, GLP-HV, GLP-H, and GLP-V showed 13.74 ± 0.49%, 35.15 ± 0.67%, 28.82 ± 1.84%, and 21.98 ± 1.84% clearance rates on DPPH radical. Furthermore, the IC_50_ of these four samples were 36.43 ± 1.79, 23.84 ± 1.78, 32.64 ± 5.07, and 35.77 ± 5.15 mg/mL, respectively, which were higher than that of Vc (0.12 ± 0.0025 mg/mL). The result demonstrates that polysaccharide with degradation had a certain scavenging ability to DPPH radical, and the treatment of H_2_O_2_ combined with Vc exhibited the strongest inhibition ability to this free radical, which may be due to its lowest molecular weight, smallest viscosity, and highest sulfate group.

Superoxide free radical is a type of reactive oxygen free radical generated in organisms, which can cause lipid peroxidation, accelerate the aging process of the whole body from the skin to the internal organs, and induce skin lesions, cardiovascular diseases, cancer, and other severe harms to human health. Figure 2e illustrates the scavenging ability of the superoxide radical of the four polysaccharides, and their scavenging activity was in the following order: GLP-HV > GLP-H and GLP-V > GLP. At concentrations ranging from 1 to 10 mg/mL, the inhibition of these four polysaccharides against the superoxide radical enhanced with increasing concentration. When the concentration of polysaccharide was 10 mg/mL, the inhibition activities on the superoxide radical from GLP, GLP-HV, GLP-H, and GLP-V were 40.87 ± 0.51, 69.96 ± 0.21, 51.54 ± 1.13, and 48.63 ± 0.82%, respectively. Moreover, the IC_50_ values of GLP, GLP-HV, GLP-H, and GLP-V were 21.48 ± 2.55, 3.56 ± 0.0028, 12.47 ± 2.56, and 13.21 ± 0.75 mg/mL, respectively, implying that GLP-HV had the best effect on the superoxide radical.

The hydroxyl radical is the most powerful reactive oxygen species (ROS), which can oxidize proteins, lipids, DNA, and sugar. The hydroxyl group contained in polysaccharide can be redox with hydroxyl radicals to remove them, thus achieving the effect of eliminating the hydroxyl radical. Figure 2f is the scavenging ability of the hydroxyl radical, and polysaccharide presented a weak scavenging ability on the hydroxyl radical compared with other free radicals. GLP, GLP-HV, GLP-H, and GLP-V displayed 22.28 ± 0.001, 19.18 ± 0.29, 16.33 ± 0.14, and 18.48 ± 0.99% hydroxyl radical scavenging activities at 10 mg/mL, respectively. The IC_50_ value of the hydroxyl radical was not calculated due to its lowest scavenging rate and was not very correlated with the polysaccharide concentration.

According to the above results, the free radical scavenging ability of the four polysaccharides declined in the order of GLP-HV > GLP-H, GLP-V > GLP. The scavenging abilities of these four polysaccharides to free radicals is in the order of superoxide radical > ferric ion > ABTS^+^ > DPPH > hydroxide radical, respectively. Furthermore, it was possible to suggest that the sulfate group, carbon group, reducing sugar content, and molecular weight were more contributing to these free radical scavenging activity of polysaccharide.

### 2.4. Cell Viability of GLP, GLP-HV, and H_2_O_2_ on HepG2 Cells and Establishment of HepG2 Cells with Oxidative Damage Model Induced by H_2_O_2_

**Figure 3:**
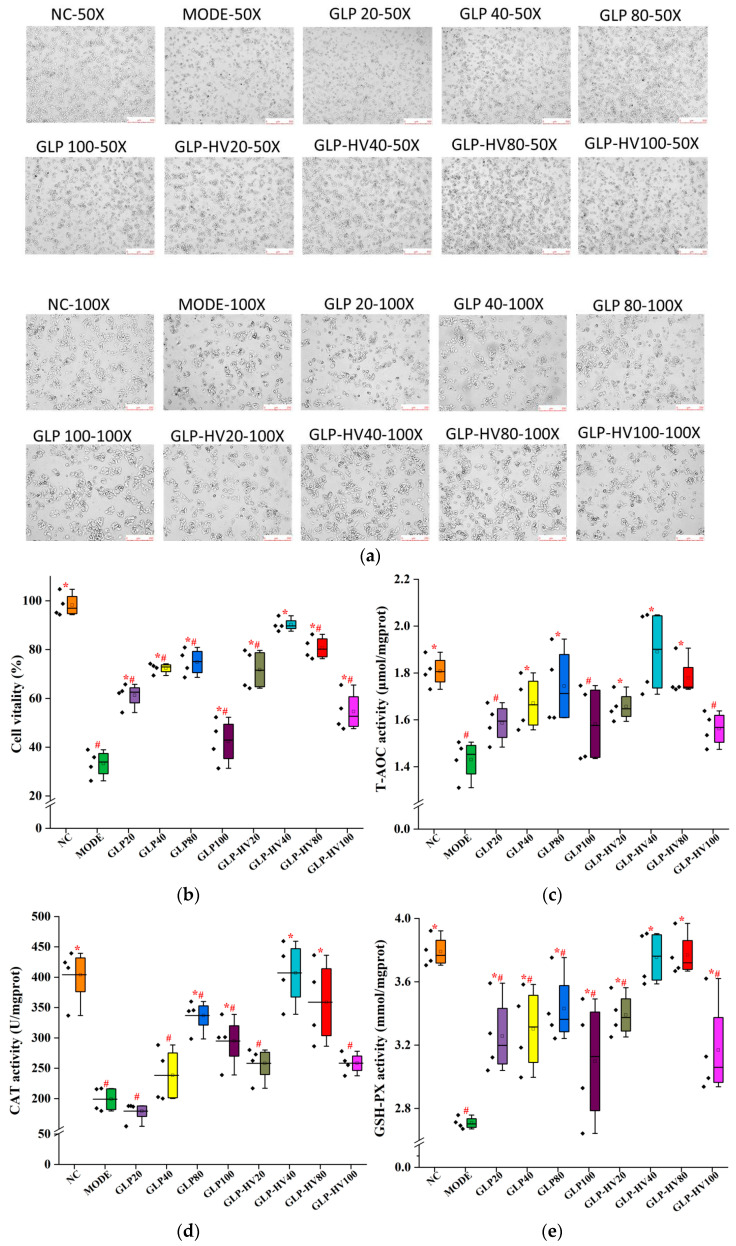

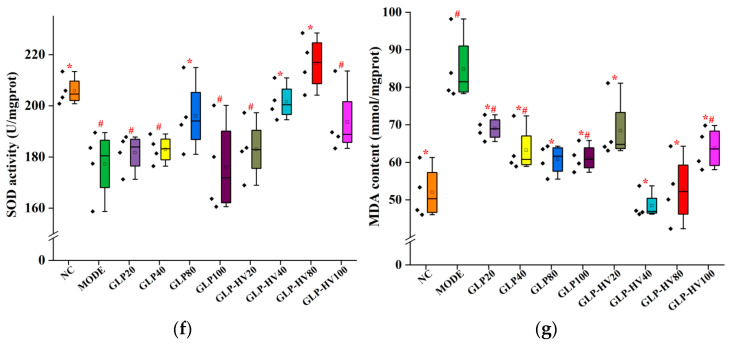
Cell viability and antioxidant activity analysis of *G. lemaneiformis* polysaccharide and its degradation products on HepG2 cells. Note: (**a**) the effect of GLP and GLP-HV on HepG2 cell morphology (inverted microscope with 50× and 100×), (**b**) the cell viability of GLP and GLP-HV on HepG2 cells, (**c**) the T-AOC activity of GLP and GLP-HV on HepG2 cells, (**d**) the CAT activity of GLP and GLP-HV on HepG2 cells, (**e**) the GSH-PX activity of GLP and GLP-HV on HepG2 cells, (**f**) the SOD activity of GLP and GLP-HV on HepG2 cells, (**g**) the MDA content of GLP and GLP-HV on HepG2 cells. “*” represents a significant difference between the other groups and the model group, “#” represents a significant difference between the other groups and the NC group, and *p* < 0.05 indicates a significant difference. The black diamond squares in (**b**–**g**) represent the value of each indicator.

H_2_O_2_, as an inducer of superoxide transformation leaked from mitochondria, is one of the main factors causing excessive oxidative stress, which is widely used to establish a cell oxidative damage model [26]. In this assay, HepG2 cells were pretreated with GLP-HV and GLP (20, 40, 60, 80, and 100 μg/mL) for 24 h, and then exposed to 100 μg/mL H_2_O_2_ for 6 h to induce a model of oxidative stress, referred to by Hu et al. [27] with some modifications. The result showed that H_2_O_2_ changed the morphology and number of cells, while the addition of polysaccharide alleviated this change and reduced cell damage (Figure 3a). Moreover, the cell viability of the model group was 33.28 ± 5.49% compared with the NC group (normal control group, without H_2_O_2_ and polysaccharide), illustrating the successful establishment of the mode of oxidative stress. As shown in Figure 3b, the treatment of 20–100 μg/mL GLP or GLP-HV for 24 h observably improved the viability of HepG2 cells (*p* < 0.05), compared with the model group (treated by H_2_O_2_ and without polysaccharide). The results disclosed that GLP-HV with 40 μg/mL exhibited the best protection for oxidative stress, and the cell viability reached up to 90.22 ± 2.64%.

### 2.5. Effects of GLP and GLP-HV on T-AOC, CAT, GSH-PX, SOD, and MDA Activity in HepG2 Cells

T-AOC refers to the ability of reducing iron ions to ferrous ions under acidic conditions, which can reflect the total antioxidant capacity of the substances. As exhibited in Figure 3c, GLP-HV (20, 40, and 80 μg/mL) and GLP (40 and 80 μg/mL) treatments were significantly enhanced compared with the mode group (1.44 ± 0.087 μmol/mgprot) (*p* < 0.05), and GLP-HV with 40 μg/mL (1.89 ± 0.18 μmol/mgprot) emerged the best total antioxidant effect, close to the control group (1.81 ± 0.065 μmol/mgprot). This result was consistent with the protective effect of cell viability.

Antioxidant-related enzymes, such as CAT, SOD, and GSH-PX, can effectively eliminate free radicals generated by oxidative stress and reduce hyperoxide to alleviate cell oxidative damage [28]. In Figure 3d–f, compared with the NC group, the activities of GSH-PX (from 3.79 ± 0.097 to 2.71 ± 0.037 mmol/gprot), SOD (from 205.87 ± 5.44 to 177.31 ± 13.37 U/mgprot), and CAT (from 403.99 ± 45.83 to 198.99 ± 19.87 U/mgprot) antioxidant enzymes were distinctly declined in the mode group, which were enhanced by GLP and GLP-HV treatment (*p* < 0.05). Among them, GLP-HV with 40 (the activities of GSH-PX, SOD, and CAT were 3.75 ± 0.17 mmol/gprot and 201.59 ± 6.97 and 407.19 ± 52.43 U/mgprot, respectively) and 80 μg/mL (3.77 ± 0.14, 216.65 ± 10.39, and 358.86 ± 67.81 mmol/gprot) expressed the best activity compared with other doses or GLP (*p* < 0.05). GLP-HV displayed excellent activity on SOD, which effectively scavenged superoxide radical, reducing the harmful substance in organisms. This was consistent with the results of the free radical scavenging ability, which verified the antioxidant capacity of GLP-HV.

MDA is a kind of harmful lipid peroxide produced by the reaction of ROS with a double bond of polyunsaturated fatty acids, and its high levels will cause oxidative damage to cells [27]. As seen in Figure 3g, the content of MDA was observably increased in the model group compared with the NC group (84.87 ± 9.21 nM/mgprot vs. 51.98 ± 6.96 nM/mgprot, *p* < 0.05). Similar to antioxidant enzymes’ activity, the level of MDA in the GLP and GLP-HV groups diminished considerably, and the impact of GLP-HV was better than that of GLP.

### 2.6. Effects of GLP and GLP-HV on ROS in HepG2 Cells

**Figure 4:**
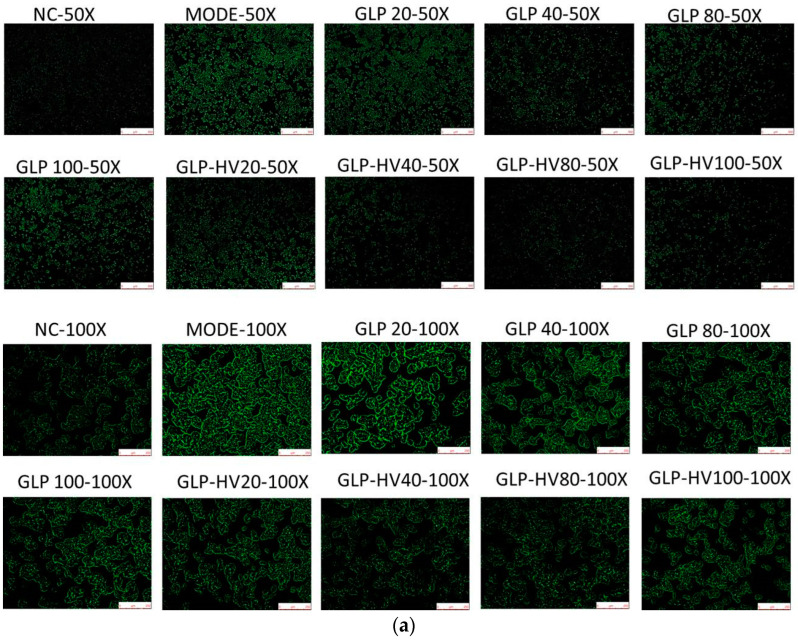

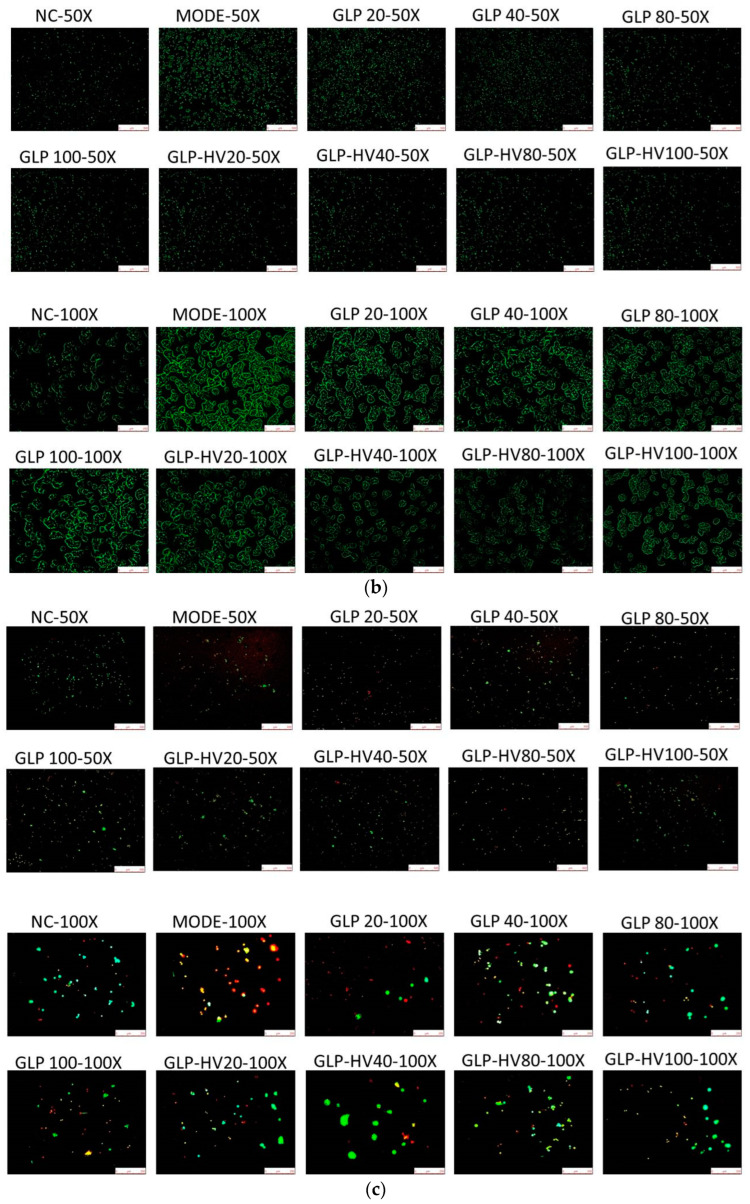
Fluorescent staining analysis of *G. lemaneiformis* polysaccharide and its degradation products on HepG2 cells. Note: (**a**) DCFH-DA fluorescent staining (50× and 100×) of GLP and GLP-HV in HepG2 cells, (**b**) fluo-3 AM fluorescent staining (50× and 100×) of GLP and GLP-HV in HepG2 cells; (**c**) AO/EB fluorescence staining (50× and 100×) of GLP and GLP-HV in HepG2 cells.

To verify the scavenging activity on ROS of GLP-HV and GLP, HepG2 cells were treated with different concentrations (20, 40, 80, 100 μg/mL) for 24 h. As presented in Figure 4a, the fluorescence intensity in the model group was markedly stronger than that of the control group, revealing that the HepG2 cells in the model group were in a state of oxidative damage [29]. GLP-HV and GLP essentially diminished the fluorescence intensity and quantity of ROS generation induced by H_2_O_2_ in HepG2 cells in a dose-dependent manner, and GLP-HV performed the best effect at 40 μg/mL. As can be seen from the above results, GLP-HV presented a strong scavenging ability on the hydroxyl radical, hence weakening the overall ROS level. It is elucidated that polysaccharide from *G. lemaneiformis* effectively attenuated excessive ROS, exhibiting excellent antioxidant activity in RAW 264.7 cells [28], which was in line with our research. The present research also exhibited raised cytotoxicity and MDA level and lower antioxidant enzymes in HepG2 cells cultured in H_2_O_2_ conditions, where the underlying mechanism may be the high content of intracellular ROS free radicals provoked by H_2_O_2_.

### 2.7. The Calcium Ion Intensity of GLP and GLP-HV in HepG2 Cells

Increasing evidence has demonstrated that a higher intracellular calcium ion concentration promotes oxidative stress and accelerates cell apoptosis. Sakanashi et al. [30] elucidated that cell death caused by H_2_O_2_ was considerably suppressed by the removal of calcium ion, which was in accord with this study. The effect of H_2_O_2_ on intracellular calcium ion is summarized in Figure 4b, and the mode group displayed greater calcium ion intensity that induced oxidative stress in HepG2 cells, compared with the NC group. A concentration-dependent trend in the scavenging action of the polysaccharide of *G. lemaneiformis* on intracellular calcium ion suffering from 100 μg/mL H_2_O_2_ is presented in Figure 4b, and GLP-HV with 40 and 80 μg/mL expressed the best inhibition on intracellular calcium ion. The results imply that the polysaccharide of *G. lemaneiformis* was able to clear intracellular calcium ion in oxidative damage cells, attenuating cell apoptosis. Additionally, degradation advanced the antioxidant activity in HepG2 cells from polysaccharide.

### 2.8. AO/EB Fluorescence Staining of GLP and GLP-HV in HepG2 Cells

Acridine orange (AO) can penetrate the intact cell membrane and embed the nuclear DNA, making it emit bright green fluorescence. AO with its positive charge can also transmit green fluorescence by binding to the negative charge of the single-stranded nucleic acid by electrostatic interaction [31]. Ethidium bromide (EB) can only penetrate the membrane of damaged cells, embedded in nuclear DNA and radiating orange fluorescence. EB cannot pass through a membrane that has the same permeability as a living one. In general, green fluorescence stands for living cells, and orange fluorescence represents dead cells [32].

H_2_O_2_ caused damage to cells, resulting in a certain degree of apoptosis and more orange fluorescence under AO/EB dye, as shown in Figure 4c, which was consistent with Figure 3a. The treatment of GLP-HV or GLP repaired the damage of cells and then reduced cells apoptosis. GLP-HV performed the best protection effect on oxidative damage cells due to its higher reactive groups and lower molecular weight and viscosity. Hu et al. [33] also explored cell survival via AO/EB staining. A higher proportion of green fluorescence indicates more living cells, whereas a stronger orange fluorescence indicates more dead cells [34].

### 2.9. Antioxidant Mechanism of GLP and GLP-HV Based on the Nrf-2/Keap-1 Signaling Pathway in HepG2 Cells

**Figure 5:**
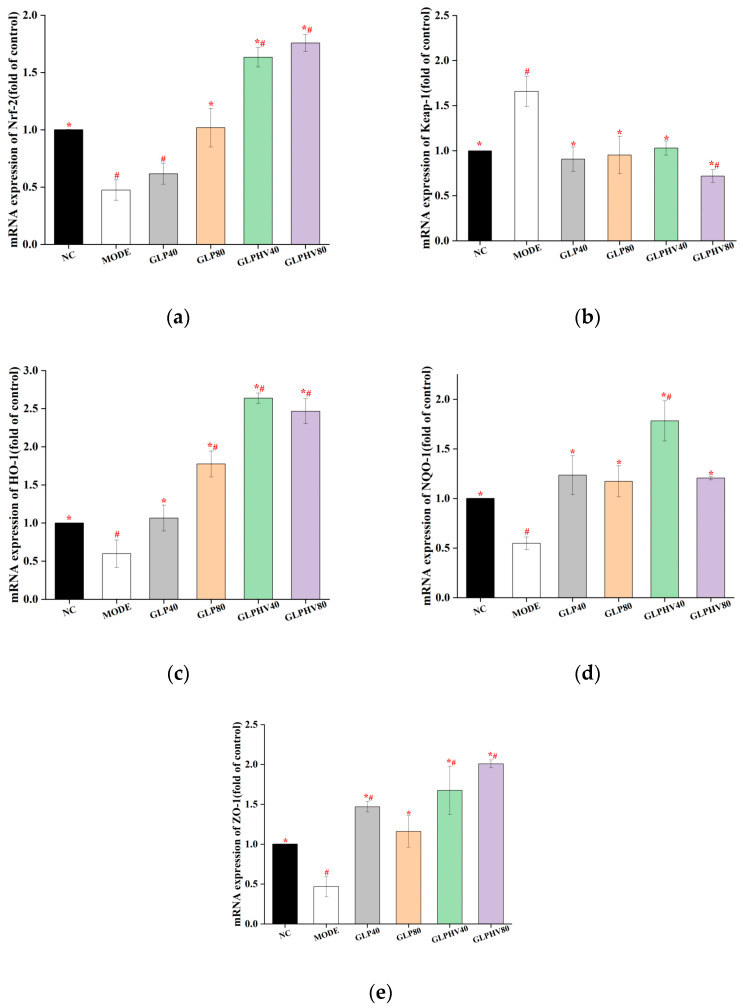
Gene analysis of the antioxidant signaling pathway of *G. lemaneiformis* polysaccharide and its degradation products on HepG2 cells. Note: (**a**) mRNA expression of the Nrf-2 gene, (**b**) mRNA expression of the Keap-1 gene, (**c**) mRNA expression of the HO-1 gene, (**d**) mRNA expression of the NQO-1 gene, (**e**) mRNA expression of the ZO-1 gene. “*” represents significant differences between the other groups and the model group, “#” represents significant differences between the other groups and the NC group, and *p* < 0.05 indicates significant differences.

To further explore the translocation of Nrf-2 into nucleus upon GLP-HV and GLP treatment, the level of Nrf-2 in each fraction was measured by real-time quantitative PCR. Figure 5a,b displays that the level of the Nrf-2 gene was augmented after being treated by GLP-HV and GLP, which inhibited the expression of Keap-1, whereas the mode group markedly weakened the Nrf-2 gene content and enhanced the level of Keap-1, compared with the NC group (*p* < 0.05). The downstream genes HO-1 and NQO-1 also were observably fortified via GLP-HV and GLP treatment, compared with the mode group (*p* < 0.05) (Figure 5c,d). Derangula et al. [21] investigated the antioxidant mechanism of probucol via the Nrf-2 signaling pathway and found that the expressions of Nrf-2 and HO-1 were strengthened and the Keap-1 level declined. Wang et al. [35] unveiled that uric acid attenuated H_2_O_2_-induced oxidative damage by the Nrf-2 pathway, which was consistent with this research. The gene of ZO-1 was regulated by the upstream genes (Nrf-2 and Keap-1), and GLP-HV and GLP upregulated the level of the gene ZO-1 compared with the model group (*p* < 0.05). It was reported that HepG2 cells treated with polysaccharide attenuated cell cytotoxicity, intracellular ROS generation, the induction of oxidative stress via the decline of the Keap-1 gene expression, and the upregulation of Nrf-2, HO-1, NQO-1, and ZO-1.

### 2.10. The Correlation between Chemical Properties and Antioxidant Mechanism of GLP-HV

**Figure 6:**
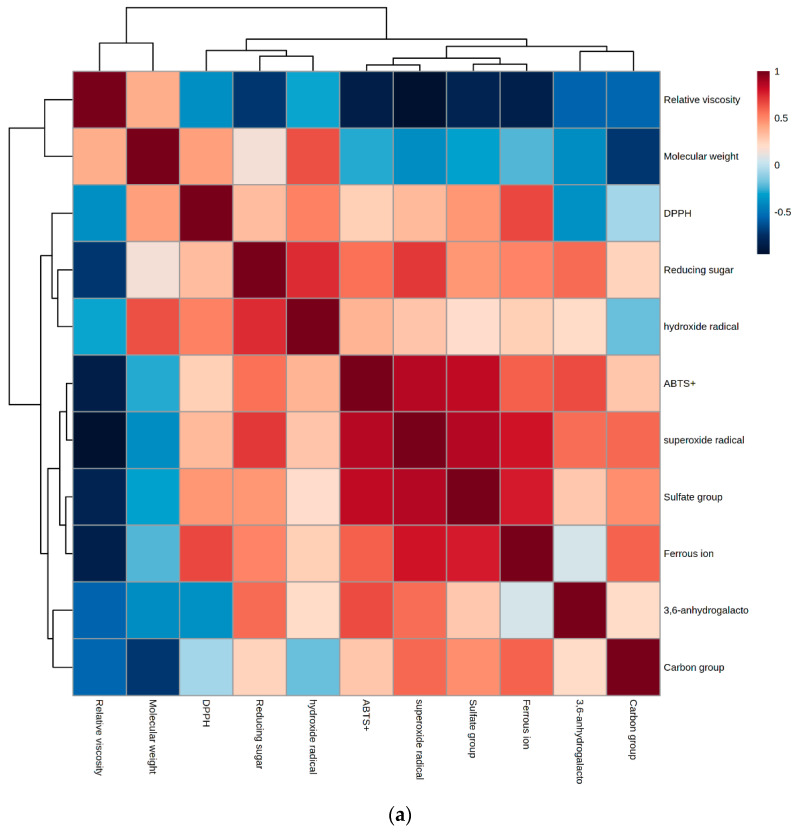

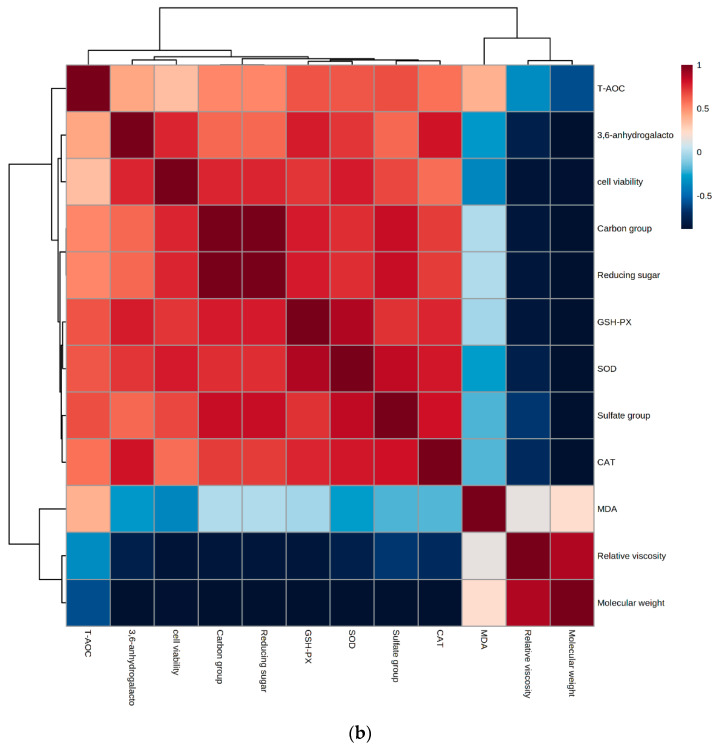

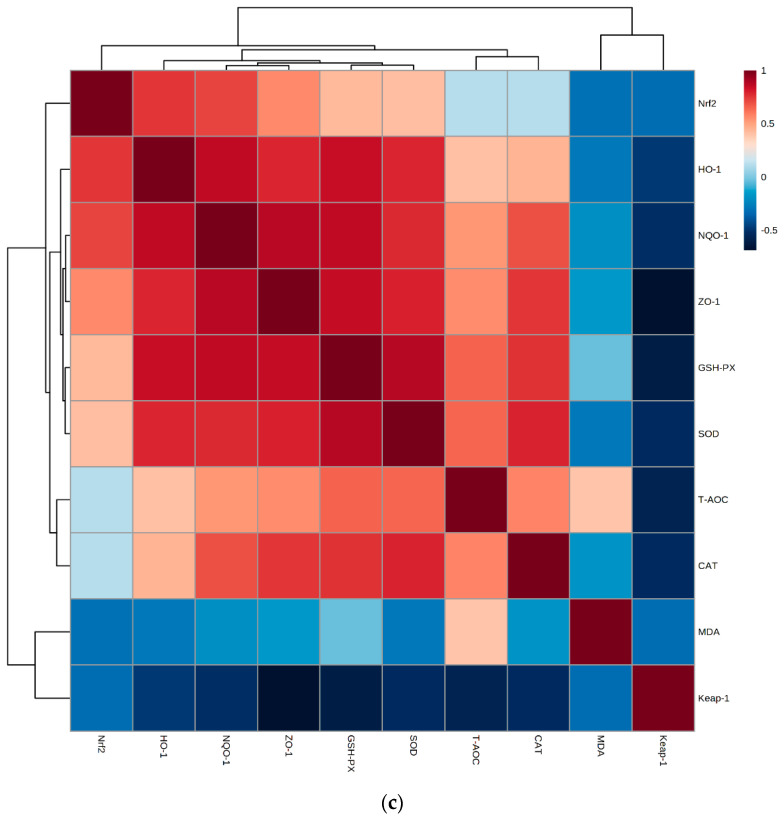
The correlation between chemical properties and the antioxidant capacity of GLP-HV. Note: (**a**) The correlation between chemical properties and the scavenging ability of free radicals from GLP-HV, (**b**) the correlation between chemical properties and the antioxidant capacity in HepG2 cells of GLP-HV, (**c**) the correlation between antioxidant indexes and the Nrf-2/Keap-1 signaling pathway in HepG2 cells of GLP-HV.

Figure 6a displays the correlation between chemical properties and the scavenging ability of free radicals from GLP-HV. The result indicated that relative viscosity was positively correlated with molecular weight and negatively correlated with other indexes. GLP-HV with extreme viscosity still had a considerable inhibitory effect on the scavenging ability of free radicals. The molecular weight expressed the negative correlation with active groups and the scavenging ability of ABTS^+^ and the superoxide radical and chelating ability of ferrous ion. In addition, 3,6-anhydrogalactose was positively correlated with the scavenging ability of these free radicals, except DPPH. Furthermore, the content of the reducing sugar and sulfate group presented an apparent positive correlation with all of the free radicals’ scavenging ability, while the level of the carbon group performed a poor correlation with DPPH and hydroxide radical. Consequently, the savaging capacity of these free radicals has a strong correlation with molecular weight, reducing sugar, viscosity, active groups. Generally speaking, high molecular weight and viscosity restrained the free radical scavenging ability of polysaccharide, while the active groups promoted the free radicals’ scavenging ability.

Figure 6b exhibits the correlation between chemical properties and antioxidant capacity in HepG2 cells of GLP-HV. The heat map illustrated that the cell viability, T-AOC, CAT, GSH-PX, and SOD ability presented a positive relationship with the level of 3,6-anhydrogalactose, reducing sugar, sulfate, and carbon groups, but displayed a negative effect with molecular weight and relative viscosity. Conversely, MDA showed the opposite result. The stronger levels of T-AOC, CAT, GSH-PX, and SOD and the weaker MDA content confirmed the higher antioxidant capacity. Hence, 3,6-anhydrogalactose, reducing sugar, sulfate, and carbon groups enhanced the antioxidant ability of GLP-HV on HepG2 cells.

To discover whether antioxidant indexes of T-AOC, CAT, GSH-PX, SOD, and MDA were correlated with the gene expression levels of the Nrf-2 signaling pathway, Spearman’s correlation was analyzed, which is shown in Figure 6c. The result reveals that the positive correlation occurred in the Nrf-2 and Keap-1 genes, which promoted the expression of HO-1, NQO-1, and ZO-1 and expressed the remarkable positive correlation. Correlation results indicated that the Nrf-2, HO-1, NQO-1, and ZO-1 genes intensified the activities of T-AOC, CAT, GSH-PX, and SOD and suppressed the content of MDA. Nevertheless, HO-1 exhibited a negative relation with T-AOC, CAT, GSH-PX, and SOD activities and played a positive relation with MDA.

Therefore, GLP-HV displayed an excellent antioxidant capacity, which was primarily attributed to the effect of reducing sugar, molecular weight, and active groups, promoting the upregulation or downregulation of genes in cells, and further altering antioxidant-related enzymes.

## 3. Discussion

The results of chemical properties showed that degradation enhanced the content of 3,6-anhydrogalactose, sulfate, and carbon groups, which may be related to the change of component proportion caused by the break of glycosidic bond. The positions of glycosidic bond breaks are different, and the exposed groups are also diverse. More sulfate and carbon groups were exposed to the break of the glycosidic bond resulting from free radicals. Additionally, the viscosity of *G. lemaneiformis* polysaccharide was obviously declined after degradation by H_2_O_2_-Vc. The mechanism of the decline in the viscosity of polysaccharide may be that Vc promoted the production of hydroxyl radicals from H_2_O_2_ [36]. The hydroxyl radical attacked the glycosidic bond of monosaccharide, making the sugar chain shorter, the molecular weight smaller, and the structure changed to a certain extent, so the viscosity also decreased. Moreover, the content of these chemical properties may be related to the antioxidant properties of polysaccharide.

Free radicals are produced by organisms in the process of metabolism, which cause oxidative stress and provoke a large number of reactive oxygen species and reactive nitrogen species free radicals [37]. Excessive reactive oxygen species can induce inflammation, disrupt the metabolism of the body, lead to damage of normal cells and tissues, accelerate oxidation and aging, and thus induce various diseases [26]. The scavenging ability of polysaccharide to free radicals reflects its antioxidant ability, and a high level of free radical scavenging ability indicates strong antioxidant ability. Three kinds of degradation polysaccharides, especially CLP-HV, exhibited a better scavenging effect on radicals than that on GLP (*p* < 0.05). Moreover, CLP-H and CLP-V performed a similar scavenging effect, both of which were stronger than GLP. The combined degradation of H_2_O_2_ and Vc can promote the breaking of glycosidic bond to a greater extent, which minimized the molecular weight of polysaccharide, enhanced the active groups, and may have stronger dehydrogenation ability to react with more free radicals. The IC_50_ values of GLP-HV on ABTS^+^, ferric ion, DPPH, and superoxide radicals were 9.62 ± 0.35, 4.97 ± 0.18, 23.85 ± 1.78, and 3.56 ± 0.0028 mg/mL, respectively, which implied that the scavenging ability of polysaccharide to these free radicals from strong to weak was as follows: superoxide radical, ferric ion, ABTS^+^, and DPPH radical. Di et al. [9] investigated the ABTS^+^ and DPPH free radical scavenging ability of GLP and verified that GLP performed excellent antioxidant activity with dose dependence between concentration and scavenging ability, which was consistent with this study. Numerous studies have revealed that polysaccharide displays stronger physiological activity after degradation or modification [13,14]. Degradation or modification may change the active groups and reduce the molecular weight of polysaccharide, resulting in the strength of its electron supply capacity. Tang et al. [15] discovered that a modified polysaccharide of *G. lemaneiformis* expressed a greater scavenging ability on free radicals, which was in accord with our results. In a previous study, the total sugar content and the reducing sugar content of GLP-HV were 98.43 ± 3.21% and 46.92 ± 2.38%, respectively [19]. The stronger scavenging ability of GLP-HV could be attributed to its highest total sugar content and the reducing sugar content, which made it the best hydrogen donor [29]. Yuan et al. [24] thought that the outstanding DPPH free radical scavenging activity of polysaccharide may be attributed to its sulfate content.

To research the antioxidant activity of GLP-HV on HepG2 cells with an oxidative damage model induced by H_2_O_2_, the cell viability, antioxidant-related enzymes, intracellular ROS, and calcium ion intensity were explored. The results explained that the polysaccharide of *G. lemaneiformis* before and after degradation presented a significant antioxidant capacity, and degradation reinforced the activity. Wen et al. [12] discovered that GLP ameliorated the levels of SOD, GSH-PX, and CAT, attenuating the MDA level, which demonstrated an outstanding antioxidant activity. This was comparable to our results. The above results indicated that the antioxidant activity may be partially attached to their ability to reinforce the activities of antioxidant enzymes in HepG2 cells and weaken the degree of intracellular oxidative stress.

ROS is formed as a natural byproduct of normal oxygen metabolism and plays an important role in cellular signaling and homeostasis [38]. ROS widely refers to free radicals and nonfree radicals derived from oxygen, including superoxide anion, H_2_O_2_, hydroxyl radical, ozone, and singlet oxygen, which have high chemical reactivity because they contain unpaired electrons [39]. High levels of ROS can cause serious damage to the cell structure, resulting in oxidative stress and promoting cell apoptosis. DCFH fluorescence probe is a simple and convenient method to detect ROS content. DCFH probe fluorescence can be oxidized to dichlorofluorescein (DCF) diacetate by ROS, and DCF can be cleaved to dichlorofluorescein by esterase, which can be observed via an inverted fluorescence microscope [29]. GLP-HV performed a better scavenging activity on intracellular ROS than GLP, which may be related with the high content of chemical properties. This result may contribute to the scavenging of hydroxyl radical and the decrease in MDA content. Fluo-3 AM is a fluorescent dye that can penetrate a cell membrane and is one of the most commonly used fluorescent probes to detect intracellular calcium ion concentration, which can accurately and swiftly reflect intracellular calcium ion intensity [40]. Fluo-3 AM can be cleaved by intracellular esterase to form fluo-3 after entering the cell, hence being retained in the cell. Fluo-3 is able to bind with calcium ion, which generates powerful fluorescence [41]. The results revealed that GLP and GLP-HV were able to clear intracellular calcium ion in oxidative damage cells, attenuating cell apoptosis. Additionally, degradation advanced the antioxidant activity in HepG2 cells from polysaccharide. In addition, oxidative stress can cause cell damage and apoptosis, which can be observed by AO/EB fluorescence staining. It was observed that *G. lemaneiformis* polysaccharide markedly alleviated the damage and apoptosis of HepG2 cells.

To further discover the antioxidant mechanism of GLP and GLP-HV based on the Nrf-2/Keap-1 signaling pathway in HepG2 cells, this passage detected the expression of the genes of Nrf-2, Keap-1, HO-1, NQO-1, and ZO-1 by real-time quantitative PCR. Nrf-2 plays a key role in oxidative stress response and is involved in the regulation of multiple genes related to oxidative stress, and is associated with a variety of antioxidant mechanisms [21]. Nrf-2 transports from the cytoplasm to the nucleus by recognizing chemical signals transmitted by oxidative and electrophilic molecules. Furthermore, it binds antioxidant reaction element sequences in numerous genes, including those encoding antioxidants, and allures their expression to enhance cell survival [35]. Activation of Nrf-2 inhibits the expression of the Keap-1, an important regulator of cellular oxidative stress response, and provokes or restrains the downstream gene level [42]. Rahman et al. [20] discovered that peptide treatment enhanced the level of Nrf-2 and HO-1, and then performed an excellent antioxidant activity. The genes of HO-1, NQO-1, and ZO-1 were regulated by the upstream genes (Nrf-2 and Keap-1), and GLP-HV and GLP upregulated the HO-1, NQO-1, and ZO-1 levels compared with the model group. Correlation results indicated that the Nrf-2, HO-1, NQO-1, and ZO-1 genes intensified the activity of T-AOC, CAT, GSH-PX, and SOD and suppressed the content of MDA. The most fundamental reason for this mechanism of action may be the high content of 3,6-anhydrogalactose and reactive groups and the low content of molecular weight and viscosity.

## 4. Materials and Methods

### 4.1. Materials and Chemicals

*G. lemaneiformis* was purchased from Nan’ao Island (Shantou, Guangdong, China); ABTS and the Frap assay kit were purchased from Beyotime Biotechnology Co., Ltd. (Shanghai, China); the T-AOC ELISA kit was bought from ZCIBIO Technology Co., Ltd. (Shanghai, China); 2,2-diphenyl-1-picrylhydrazyl (DPPH) was from Macklin Biochemical Technology Co., Ltd. (Shanghai, China); the superoxide anion radical assay kit was from Geruisi Biochemical Technology Co., Ltd. (Suzhou, China); RIPA, fluo-3 AM, and CCK-8 were purchased from Solarbio Biotechnology Co., Ltd. (Beijing, China); BCA, SOD, MDA, CAT, and the GSH-PX assay kit were purchased from Nanjing Jiancheng Biotechnology Co., Ltd. (Nanjing, China); 2′,7′-dichlorofluorescein diacetate (DCFH-DA) was purchased from Shanghai Yuanye Biotechnology Co., Ltd. (Shanghai, China); and the AO/EB double fluorescence staining kit belonged to Phygene Biotechnology Co., Ltd. (Fujian, China).

### 4.2. Extraction and Degradation of G. lemaneiformis Polysaccharide

The extraction and degradation methods of *G. lemaneiformis* polysaccharide are described in previous studies [18].

*G. lemaneiformis* polysaccharide was extracted with hot water and the ethanol precipitation method; the extracting conditions were as follows: the ratio of material and liquid was 1:45 (*w*:*v*), ultrasonic-assisted extraction was performed for 30 min, extraction was conducted at 90 °C for 4 h, and finally, dialysis and freeze-drying proceeded. The sample was prepared, named GLP.

GLP was degraded by 18.7 mM Vc and H_2_O_2_ at 56 °C for 0.5 h, and the degradation reaction was terminated by adjusting the pH to neutral, and was precipitated with ethanol, and finally, dialysis and freeze-drying were performed. The sample was prepared, named GLP-HV. GLP was degraded by H_2_O_2_ alone, called GLP-H. GLP was degraded by Vc alone, named GLP-V.

### 4.3. The Content of 3,6-Anhydrogalactose of GLP and GLP-HV

The content of 3,6-anhydrogalactose was determined by the resorcinol colorimetry method, which was referred to by Ferreira et al. [43] with slight modification.

Resorcinol reserve solution: 150 mg resorcinol was added to 100 mL pure water, stored in a brown bottle at 4 °C, used within 1 week.

Resorcinol–acetal working solution preparation: 9 mL resorcinol solution, 1 mL 0.04% acetal solution, and 100 mL concentrated hydrochloric acid mixed evenly, used only within the day.

Fructose standard curve: 0.5 mg/mL fructose standard solution was prepared, and 0, 0.1, 0.2, 0.3, 0.4, and 0.5 mg/mL fructose standard solutions were respectively placed in ice water bath for 5 min. An amount of 5 mL of resorcinol–acetal working solution was added and reacted at 80 °C for 15 min. Absorbance was measured at 554 nm after 2 min of ice water bath. A standard curve was drawn with fructose concentration as the abscissa and absorbance as the ordinate.

Sample determination: the operation steps were consistent with the standard curve, calculated by the sample fructose concentration, multiplied by the correction coefficient 1.087, which can be obtained with 3,6-anhydrogalactose content.

### 4.4. The Content of Sulfate and Carbon Groups of GLP and GLP-HV

The content of the sulfate group was determined by BaCl_2_-gelatin method referred to by Han et al. [44] with a little revision.

Standard curve: K_2_SO_4_ was precisely weighed and dissolved with 1 mol/L HCl to prepare a 0.6 mg/mL sulfuric acid standard solution. Standard solutions with 0, 0.12, 0.24, 0.36, 0.48, 0.6, 0.8, and 1.2 mg/mL were respectively put into the test tube, and 3.8 mL 3% trichloroacetic acid and 1 mL BaCl_2_-gelatin solution were added to each tube, respectively, and placed at room temperature for 15 min. Absorbance was measured at 360 nm, denoted as A_1_. BaCl_2_ was replaced by 1 mL gelatin solution, and the absorbance was measured by the same method, denoted as A_2_; the standard curve was drawn with sulfate group mass (mg) as abscissa and absorbance difference A_1_–A_2_ as ordinate.

Sample determination: a 0.05 g sample was weighed, 10 mL of 1 mol/L HCL solution was added and hydrolyzed at 100 °C for 6 h, 0.2 mL of hydrolyzed sample was taken, and the experiment was carried out according to the determination method of the standard curve.

The content of carbon group was operated as the kit instruction.

### 4.5. Viscosity Determination of GLP and GLP-HV

The viscosity was determined using a UbbeloHde viscometer [45]. The samples were formulated as 1, 2, 3, 4, and 5 mg/mL; the time of sample passing through the viscometer was recorded at 25 °C; and the determination was repeated three times.

### 4.6. Scavenging Ability of GLP and GLP-HV on Free Radicals

#### 4.6.1. Scavenging Ability of GLP and GLP-HV on ABTS^+^

An amount of 10 μL GLP or GLP-HV was added to a 96-well plate, then 200 μL ABTS working solution was added, and the mixture was oscillated. After incubation at room temperature for 2 to 6 min, the OD value was determined at a 734 nm wavelength. Vc was the control, and pure water was blank. The scavenging ability of GLP and GLP-HV on ABTS^+^ was calculated by the following formula:

ABTS^+^ scavenging rate (%) = (A_0_ − A)/A_0_ × 100 (1)

Note: A: absorbance values of GLP, GLP-HV, and Vc; A_0_: absorbance value of pure water.

#### 4.6.2. Chelating Capacity of Ferrous Ion of GLP and GLP-HV

The chelating capacity of ferrous ion was determined using a Frap (ferric ion reducing antioxidant power) kit. An amount of 5 μL of GLP or GLP-HV was added to a 96-well plate, then a 180 μL Frap working solution was added, and the mixture was oscillated. After incubation at 37 °C for 3 to 5 min, the OD value was determined at a 539 nm wavelength. Vc was the control, and pure water was blank. The chelating rate of the ferrous ion of GLP and GLP-HV was calculated by the following formula:

Chelating rate of ferrous ion (%) = (A − A_0_)/A_0_ × 100 (2)

Note: A: absorbance values of GLP, GLP-HV, and Vc; A_0_: absorbance value of pure water.

#### 4.6.3. Scavenging Ability of GLP and GLP-HV on DPPH

The scavenging ability of DPPH free radical was referred to the method [37] and modified slightly. An amount of 0.004 g of DPPH was accurately weighed, dissolved in anhydrous ethanol, and kept at a volume of 100 mL. Amounts of 20 μL of sample and 180 μL of DPPH were added to a 96-well plate after incubation at room temperature for 30 min without light, and the OD value was determined at a 517 nm wavelength. Vc was the control, and anhydrous ethanol was blank. The scavenging ability of GLP and GLP-HV on DPPH was calculated by the following formula:

DPPH scavenging rate (%) = (A_1_ − A_2_)**/**(A_3_ − A_4_) × 100 (3)

Note: A_1_: 20 μL GLP/GLP-HV/Vc + 180 μL DPPH; A_2_: 20 μL GLP/GLP-HV/Vc + 180 μL anhydrous ethanol; A_3_: 20 μL anhydrous ethanol + 180 μL DPPH; A_4_: 200 μL anhydrous ethanol.

#### 4.6.4. Scavenging Ability of GLP and GLP-HV on Superoxide Anion Radical

The scavenging ability of superoxide anion radical was referred to Tang et al. [15] and had a small modification. An amounted 10 μL of GLP or GLP-HV was added to a 96-well plate, then a working solution was added, and the mixture was oscillated. After incubation at 37 °C for 10 min, the OD value was determined at a 570 nm wavelength. Vc was the control. The scavenging ability of GLP and GLP-HV on DPPH was calculated by the following formula:

Superoxide anion radical rate (%) = (1 − (A_1_ − A_2_))/A_0_ × 100 (4)

Note: A_1_: 10 μL GLP/GLP-HV/Vc + 75 μL reagent I + 80 μL reagent II + 20 μL reagent III+ 15 μL reagent IV; A_2_: 10 μL GLP/GLP-HV/Vc + 95 μL reagent I + 80 μL reagent II + 15 μL reagent IV; A_0_: 85 μL reagent II + 20 μL reagent III + 15 μL reagent IV.

#### 4.6.5. Scavenging Ability of GLP and GLP-HV on Hydroxyl Radical

The hydroxyl radical scavenging rate was determined by a Fenton system [29]. After mixing 6 mmol/L ferrous sulfate and 6 mmol/L hydrogen peroxide 2.5 mL each, 6 mmol/L salicylic acid 7.5 mL was added. After fully mixing, the system was placed in 37 °C water bath for reaction for 15 min. The absorbance A_0_ was measured at a 510 nm wavelength. An amount of 1 mL of sample was added to the system, and the absorbance A_1_ was measured at 510 nm after reaction with water bath at 37 °C for 15 min. Vc was the control, and pure water was blank. The scavenging ability of GLP and GLP-HV on hydroxyl radical was calculated by the following formula:

Hydroxyl radical scavenging rate (%) = (A_0_ − A_1_)/A_0_ × 100 (5)

### 4.7. Cell Culture

HepG2 (CX0004) cells were purchased from Boster Biological Technology Co., Ltd. (Wuhan, China), which were cultured with MEM medium containing 10% FBS and 1% penicillin–streptomycin in an incubator with 5% CO_2_ at 37 °C [39].

### 4.8. Cell Viability Assay

The cells were seeded into a BioClean 96-well plate at 5 × 10^3^ cells/well and cultivated for 24 h. After the medium was discarded, the cells were washed twice with PBS and added with different concentrations of samples (20, 40, 80, and 100 μg/mL) for 24 h cultivation. Then 100 μg/mL H_2_O_2_ was added for 6 h cultivation according to the literature with a few modifications [26]. The viability of the cell was measured with a CCK-8 assay. Amounts of 10 µL of CCK-8 solutions were added to each well and cultured in an incubator for 2 h, and absorbance was finally measured at 450 nm. Cell viability was calculated by the following formula:

Cell viability (%) = (A − A_blank_)**/**(A_0_ − A_blank_) × 100 (6)

Note: A: absorbance values of cells, CCk-8, GLP, and GLP-HV; A_blank_: absorbance values of medium and CCk-8; A_0_: absorbance values of cells and CCk-8.

### 4.9. Analysis of Antioxidant Indexes of GLP and GLP-HV in HepG2 Cells

The antioxidant activity of GLP and GLP-HV to HepG2 cells was mainly through T-AOC, CAT, GSH-PX, SOD, and the MDA assay kit [46], which was operated according to the kit instructions. The protein content of HepG2 cells was detected by the BCA assay kit.

### 4.10. Effects of GLP and GLP-HV on ROS in HepG2 Cells

The intracellular reactive oxygen species (ROS) level was determined by the 2′,7′-dichlorofluorescein diacetate (DCFH-DA) fluorescence probe assay [28]. The solution of DCFH-DA (5 μM) was added to HepG2 cells and incubated at 37 °C for 10 min. The supernatant was discarded, and cells were washed with PBS three times. The images were observed under an inverted fluorescence microscope (Leica Application Suite X 3.4.2.18368, Shanghai, China) without light.

### 4.11. The Calcium Ion + Intensity of GLP and GLP-HV in HepG2 Cells

Fluo-3 AM was used to determine the level of intracellular calcium ion in HepG2 cells, and referred to the literature with some modification. The solution of Fluo-3 AM (5 μM) was added to HepG2 cells and incubated at 37 °C for 15 min. The supernatant was discarded, and cells were washed with PBS twice. The images were observed under an inverted fluorescence microscope without light.

### 4.12. AO/EB Fluorescence Staining of GLP and GLP-HV in HepG2 Cells

The method of AO/EB fluorescence staining was according to the method with a little modification [44]. After the cells were digested with 25% trypsin, the cells were suspended with 100 μL PBS, 2 μL AO/EB solutions were added, and the supernatant was discarded by centrifugation at 500× *g* for 5 min. The cells were suspended again with 50 μL AO/EB dilution buffer, and then 2 μL AO/EB solutions were added. At last, 5 μL cell suspensions were dropped on the slide, and a cover slide was gently put on it, which was observed under an inverted fluorescence microscope without light.

### 4.13. Real-Time Quantitative PCR Detection

The information of the gene primer design is presented in Table 2. The cells were washed twice with PBS and lysed with a TRIzol reagent. After shaking evenly, the cells were placed on ice for reaction for 10 min, and the RNA was extracted. RNA integrity was detected by 2% gel, and RNA concentration was detected by NanoDrop 2000. The total RNA was reversely transcribed into cDNA according to the instructions of TaKaRa PrimeScript^TM^ RT Reagent Kit with gDNA Eraser. Finally, real-time quantitative PCR was performed.

### 4.14. Statistical Analysis

Data are expressed as means ± standard (SD). Duncan’s multiple range test was applied to identify differences between the mean values for each group by IBM SPSS software (version 22, Palo Alto, CA, USA). *p* < 0.05 was considered to represent statistical significance. Figures were finished by Origin 8.5.

## 5. Conclusions

In this research study, the molecular weight and viscosity of polysaccharide were greatly lowered, and 3,6-anhydrogalactose and sulfate and carbon groups were observably enhanced after degradation via H_2_O_2_-Vc. GLP-HV performed a better scavenging ability on superoxide radical, ferric ion, ABTS^+^, and DPPH radicals compared with GLP. This study explored the antioxidant activity of GLP and GLP-HV supplementation on H_2_O_2_-induced HepG2 cells. The viability of oxidative damage cells was remarkably strengthened via GLP and GLP-HV, particularly the latter. Additionally, CAT, SOD, and GSH-PX levels were found to be fortified considerably, while MDA content declined. Moreover, the fluorescence results implied the antioxidant capacity of GLP and GLP-HV on HepG2 cells, which substantially diminished ROS and calcium ion intensity. The gene expressions of Nrf-2, HO-1, NQO-1, and ZO-1 were enhanced, and Keap-1 was inhibited. Consequently, GLP and GLP-HV performed an excellent antioxidant capacity attributed to the Nrf-2/Keap-1 signaling pathway. Furthermore, GLP-HV showed that the better effect may be due to the higher content of 3,6-anhydrogalactose, sulfate group, carbon group, and lower viscosity through the correlation analysis of a heat map.

## Figures and Tables

**Table 1:** The content of 3,6-anhydrogalactose, sulfate group, and carbon group of *G. lemaneiformis* polysaccharide and its degradation products.

Content	GLP	GLP-HV	GLP-H	GLP-V
3,6-Anhydrogalactose (%)	35.69 ± 0.34 ^c^	37.18 ± 0.48 ^b^	34.04 ± 0.77 ^d^	43.98 ± 1.07 ^a^
Standard curve equation of 3,6-anhydrogalactose	Y = 1.5813 X + 0.1831 *R*^2^ = 0.9921			
Sulfate group (%)	4.22 ± 0.08 ^c^	7.53 ± 0.0.05 ^a^	5.38 ± 0.25 ^b^	5.48 ± 0.13 ^b^
Standard curve of equation sulfate group	Y = 0.60024 X + 0.2273 *R*^2^ = 0.9981			
Carbon group (μmol/g)	3.31 ± 0.19 ^b^	4.22 ± 0.32 ^a^	4.32 ± 0.14 ^a^	4.41 ± 0.02 ^a^

Note: a, b, c, and d represent a significant difference among groups on the same index, and *p* < 0.05 indicates a significant difference.

**Table 2:** Primers used for RT-PCR assay.

Gene	NCBI Reference Sequence	Primer Sequences	
		Forward	Reverse
GAPDH	NM_001289726.1	GGAGAAACCTGCCAAGTATGATGAC	GAGACAACCTGGTCCTCAGTGTA
Nrf-2	NM_010902.5	CAGTGCTCCTATGCGTGAATCCC	TGCCCTAAGCTCATCTCGTGTGA
Keap-1	NM_016679.4	CGAAGAGGCGGCAGAAGAAG	GACGCTCCAGGGCTATGACA
HO-1	NM_010442.2	ACCGCCTTCCTGCTCAACATTG	CTCTGACGAAGTGACGCCATCTG
NQO-1	NM_008706.5	GTCTGGAAACCGTCTGGGAGGA	GCCCACAGAGAGGCCAAACTTG
ZO-1	NM_009386.2	GGTGCCCTGAAAGAAGCGAT	CTGACAGGTAGGACAGACGA

## Data Availability

All data are included in the manuscript.
